# Self-Rated Health and Psychological Distress among Emerging Adults in Italy: A Comparison between Data on University Students, Young Workers and Working Students Collected through the 2005 and 2013 National Health Surveys

**DOI:** 10.3390/ijerph18126403

**Published:** 2021-06-13

**Authors:** Isabella Giulia Franzoi, Fabrizio D’Ovidio, Giuseppe Costa, Angelo d’Errico, Antonella Granieri

**Affiliations:** 1 Department of Psychology, University of Turin, 10124 Turin, Italy; antonella.granieri@unito.it; 2 Department of Neurosciences “Rita Levi Montalcini”, University of Turin, 10126 Turin, Italy; fabrizio.dovidio@unito.it; 3 Department of Clinical and Biological Science, University of Turin, 10043 Orbassano, Italy; giuseppe.costa@unito.it; 4 Department of Epidemiology, Azienda Sanitaria Locale TO3, 10095 Grugliasco, Italy; angelo.derrico@epi.piemonte.it

**Keywords:** emerging adulthood, university students, young workers, health, distress

## Abstract

Background. The present study aimed at comparing self-reported physical health and mental health among university students, workers, and working students aged between 19 years and 29 years. Method. Using data from National Health Surveys held in 2005 and 2013, a cross-sectional study was conducted on 18,612 Italian emerging adults grouped into three groups: university students, workers, and working students. The odds ratios of self-reported anxiety or depression, poor general health, and poor mental health and physical health (as assessed through SF-12) were estimated through logistic regression models adjusted for potential confounders. Results. Compared with workers, students showed an increased risk of anxiety or depression and a lower risk of poor general health. Students and working students showed an increased risk of reporting weak mental health compared with that in workers, while students displayed a lower risk of poor physical health. Significant differences were not found between the 2005 and 2013 surveys. Conclusions. These results are of considerable importance for psychologists as well as educational and occupation-based institutions for planning prevention programs and clinical interventions.

## 1. Introduction

“Emerging adulthood” (EA) [1] is a transitional age between adolescence and adulthood (approximately in the third decade) characterized by emancipation, financial self-sufficiency, and choices about career and intimate relationships. In this phase of development, people prepare for their adult lives, explore different possibilities and make decisions that will define who they are in the outside world and in their own minds [2,3].

For some young people, this phase coincides with the years of university education. Entering higher education implies opportunities and risks, experiencing tasks such as transfer, performance demands, changes in living conditions and lifestyles, as well as dealing with new social and educational contexts. Recently, educational systems worldwide have shown increased concern for the mental health and emotional wellbeing of university students [4], highlighting especially depression, anxiety, suicide risk, and drug addiction [5,6,7,8,9,10,11,12,13].

For those who do not enter higher education, EA includes the school-to-work transition period. Employment can have a protective impact on young workers’ psychological wellbeing by promoting their emancipation. However, entering the workforce can also be demanding and can compromise mental health: young workers seem to experience relatively high levels of psychological distress, workplace bullying, and addictive behaviors [14] compared with older workers. They seem to suffer from greater work-related distress and to be exposed to more psycho-social stressors, such as harassment, low control of employment status, and relational conflicts with colleagues [15,16]. At the same time, psychological distress was detected in 32.2–72.9% of university students [17,18,19].

Indeed, psychological distress seems to be connected to other factors. In particular, females seem to experience greater distress, mostly connected to gender roles and social expectations [20,21]. Moreover, it is strongly related to perceived physical and mental health status. On the one hand, university students often report a significant negative impact of psychological distress on their physical and mental health [22,23]. On the other hand, students’ physical health seems to be a significant predictor of psychological distress [24], and physical health problems arising during EA along with connected health behaviors can result in different levels of psychological distress [25,26].

Some studies have also investigated psychological distress in university students compared with that of their age-matched working peers. Research data have mostly shown that university students experience a higher prevalence of psychological distress [7,27,28,29], but such results are controversial [30,31].

Psychological distress in university students is a matter of great concern for educational and occupational systems because students experiencing greater psychological distress show a higher risk of academic failure and dropping out of university [32,33]. Simultaneously, the distress experienced by workers is a major challenge for maintaining occupational health and safety [34].

However, only a few studies in Italy have focused on the distress and quality of life in people experiencing EA [35,36,37,38,39], and no study has investigated differences in mental health and physical health between university students and their working peers in Italy.

Thus, we decided to investigate perceived anxiety and depression, as well as self-rated general, physical and mental health, among 19–29 years old university students, workers, and working students in Italy, to detect potential differences connected not to this development stage, but rather to the specific declinations of EA according to academic and occupational conditions. A secondary aim was to examine possible differences in the effect of the student/working status on health before and after the 2008 financial and socioeconomic crisis by comparing data from a National Health Survey (NHS) conducted in 2005 and 2013.

## 2. Materials and Methods

### 2.1. Ethical Approval of the Study Protocol

A NHS is included in the Italian National Statistical Program (PSN code: IST-01426 for NHS 2005 and IST-02067 for NHS 2013) and was approved by the Italian Presidency of the Council of Ministers. No approval from an Ethics Committee was requested for this study, as it was conducted on anonymized public data, made freely available by the Italian National Institute of Statistics (ISTAT), Rome. Written informed consent was obtained from all participants.

All research procedures were conducted in accordance with the ethical standards of the committees responsible for human experimentation (institutional and national) and with the Helsinki Declaration of 1975, as revised in 2013.

### 2.2. Participants

The study population comprised 19–29 years old young adults recruited in 2004–2005 (*N* = 10,673) and 2012–2013 (*N* = 7939) in Italian NHSs undertaken by the Italian National Institute of Statistics. Participants were divided into three groups. The university students group comprised undergraduate and postgraduate students, whether they were full-time or part-time students who declared no working activity. The workers group comprised young adults claiming to be working and not to be studying at the time of the survey, regardless to their level of education. Finally, the working students group comprised undergraduate and postgraduate students who declared a concurrent working activity. Each NHS is representative of the Italian population and aims to investigate self-reported health status, lifestyle habits, social conditions, health conditions, and use of health services through a detailed questionnaire administered to each member of 60,000 Italian families, randomly sampled and distributed in almost 1500 municipalities of different population sizes. The percentage of people who participated was 83% for the NHS carried out in 2004–2005 and 82.3% for the NHS undertaken in 2012–2013.

### 2.3. Outcome Measures

We considered four health outcomes at the time of interview.

The first health outcome was self-reported anxiety or depression, but only if diagnosed by a physician (dummy variable was “yes” or “no”). The prevalence of depression and anxiety was documented in the same category in the NHS in 2004–2005, so depression and anxiety were considered jointly in the main analysis.

Two other outcomes were represented by the Mental Component Score (MCS) and Physical Component Score (PCS) of the Short Form-12 Health Survey (SF-12) [40,41]. Each score ranges between 0 and 100, whereby 0 indicates the lowest level and 100 indicates the highest level of health. We created a dichotomized variable for both the MCS and the PCS scales, considering scores higher than the first decile vs. lower.

The fourth health outcome was measured by a general self-rated health scale. This comprised five categories (“very good”, “good”, “fair”, “poor”, “very poor”) dichotomized as poor health vs. good health. We considered the intermediate category of “fair” as denoting poor health.

### 2.4. Statistical Analyses

With regard to descriptive statistics, the chi-square test, Fisher’s exact test, and Kruskal–Wallis equality-of-populations rank test were carried out where appropriate. The association between student/working status and the four health outcomes considered (anxiety/depression, MCS < 10 percentiles, PCS < 10 percentiles, poor health) was estimated using logistic multivariable regression models adjusted for sex, age, Body Mass Index (BMI), current sporting activity, current smoking, economic condition (“good”, “adequate”, “scarce”, “totally inadequate”), year of survey (2005 or 2013), and geographic region of Italy (Northern, Central and Southern/Insular).

## 3. Results

Men (54%) constituted most of the study population. The median age was 25 years. Significant differences between workers, students, and working students were found for sex, age, economic conditions, year of survey, BMI, smoking, sporting activity and Italian geographic region (Table 1).

Table 2 shows the descriptive statistics among workers, students, and working students for the main health outcomes investigated.

The percentage of the population with a medical diagnosis of anxiety or depression, as well as poor health, was 1% and 10%, respectively. No significant differences were detected between working status and anxiety or depression. Significant differences were reported for perceived health, MCS score, and PCS score.

Multivariate analysis showed among students, compared with that in workers, a higher risk of anxiety or depression (odds ratio (OR) = 1.48, 95% confidence interval (CI) = 1.02–2.17), and of a low MCS (1.63, 1.45–1.83), as well as a lower risk of poor general health (0.87, 0.77–0.99) and low PCS (0.72, 0.63–0.82) (Table 3).

Working students had a significantly higher risk of a low MCS (OR = 1.68, 95% CI = 1.31–2.17), whereas the other outcome measures were not associated.

Students and working students showed a similar increased risk of having a low MCS (OR = 1.63, 95% CI = 1.45–1.83, and 1.68, 1.31–2.17, respectively) compared with that in workers. For a low PCS, only students reported a significantly lower OR (0.72, 95% CI = 0.63–0.82) compared with that of workers.

An analysis stratified by the year of survey (2005 or 2013) was undertaken to assess the effect of student/working status on health before and after the 2008 financial and socioeconomic crisis: relevant differences between the two periods were not observed. An interesting difference was a higher risk of a low MCS for students and working students (vs. workers) in 2013 (OR = 1.77, 95% CI = 1.50–2.10, and 1.78, 1.22–2.59, respectively) compared with that in 2005 (1.51, 1.28–1.79 and 1.59, 1.12–2.25, respectively), although interactions were not significant (*p* = 0.409 for students and *p* = 0.762 for working students).

## 4. Discussion

We wished to explore perceived mental and physical health, self-rated general health, and anxiety or depression among 19–29 years old Italian emerging adults, and to detect potential differences between the experience of university students, workers, and working students.

We found significant differences between the three groups in all sociodemographic and behavioral covariates. Workers were mostly males (59.1%), while students and working students were mostly females (respectively 55.9% and 57.6%). Since our total sample showed a majority of men, these results could be associated with a gender gap upon entering the workforce or university. This finding merits further investigation, for example, on the potential social pressures of the basis of this choice. Students showed the lowest median age (22 vs. 26 years for both workers and student workers), which suggested a delayed entry into a career. It would be interesting to explore if young Italians experience the same age and sex-based trajectories in the initial labor market experienced by other young Europeans [42].

Most workers came from northern Italy (48.7%), as did working students (47.7%), while most students came from Southern Italy (50.4%). These data seem to be connected to a lack of job opportunities for young adults in Southern Italy [43,44,45] that could have led them to continue their studies [46].

BMI was normal in all three groups, but significantly higher among workers, of whom more than one out of five were overweight or obese. Most emerging adults were non-smokers, with a higher percentage of smokers among workers (33.7%) than that among working students (21.2%) and students (18.4%). Most participants were involved in physical activity, but workers (61.5%) less than students (75.6%) and working students (76.9%). EA can be associated with unhealthy lifestyles [47,48,49]. Research has shown that university students show bad eating habits and low physical activity [50,51], but it seems that young workers are more at risk to the onset of unhealthy behaviors.

For what concerns the main health outcomes investigated, we expected that working students would experience the worst physical and mental health, whereas young workers would show the best.

Our sample showed a lower prevalence of distress than that observed in other research [5,13]. In our sample, the prevalence of anxiety or depression was 1%: this could have been because these disorders were self-reported. Indeed, some emerging adults who experience anxious and depressive symptomatology may not be aware of their problems, do not know what to call them, and do not turn to physicians for a diagnosis. Furthermore, we found low levels of poor perceived general health (10.1%). On the contrary, De Waure and colleagues studied a cohort of Italian university students and showed that most of them experienced good perceived health, but a higher proportion of young adults were dissatisfied about their health status [52]. This discrepancy could be related to differences in enrolment because the study by De Waure and coworkers (2015) comprised only students from a private Catholic university, and the percentage of participating students was much lower (~70%) than that of the present study, thereby suggesting selection of a less healthy study sample.

We found better mental and physical health than that documented in a cross-validation study of SF-12 for an Italian cohort [53], although that study involved an older population (18–44 years). These results focus on common characteristics of this developmental stage and are in accordance with other research, suggesting that young adults may experience a feeling of instability and flux, as well as many emotional, economic, and social conflicts, while they face new commitments connected to relationships, career, and academic life [54,55]. In some cases, the ability to make so many choices concerning their actual and future life can be compromised if emerging adults have experienced difficulties in their identity development and they cannot identify their specific qualities and capacities [3]. For some emerging adults, these challenges can be too difficult to face, and can lead to high levels of distress.

For what concerns the hypothesized difference between the three groups according to their students/workers status, we found that being a student was associated significantly with higher levels of anxiety or depression and poor perceived health [56,57]. These results should be regarded cautiously. Indeed, the differences between the three groups were small, even if significant. Moreover, we have to take into account that the differences in the experience of students and workers should be connected to the fact that students can be distressed because of an uncertain future, whereas for workers current time is the main source of conflicts. Thus, these observations merit further investigations, in particular as regards which elements of physical health and mental health are taken into account by emerging adults when considering their global health status. Indeed, it could be that young adults consider physical conditions more than mental conditions when defining their health status.

Moreover, we found significant differences for the MCS and PCS. Students showed a greater risk of a low MCS compared with that of workers, and this scenario was even more pronounced among working students. In contrast, workers seemed to have a greater risk of a low PCS compared with that of the other two groups.

Firstly, these results suggest that care and attention should be given to working students, because they seem to experience the highest risk of weak mental health. Their level of distress may be worsened by the need to balance academic and occupational demands as well as by multifaceted risk factors. Indeed, when working students perceive their work demands as interfering with university (e.g., if they are exhausted from a working day but must study for examinations or assignments), they are likely to experience negative tension due to role conflict [58], which can lead to physical (e.g., headache, fatigue) or psychological (e.g., excessive worry, anxiety) strain [59].

Moreover, our results suggest that, in Italy, university students experience greater distress than that experienced by their working peers (who are not concurrently students). These results are controversial with respect to the literature [7,27,28,29]. However, our results are consistent with literature which states that, in university students, emotional wellbeing is more of a concern than physical wellbeing [60,61]. This observation seems to demonstrate that the university experience can be worthwhile and stressful, and that not every student may succeed in facing the associated challenges. Previous research has shown that not only academic challenges have an impact on the psychological distress felt by university students, but that other factors should be considered, such as social interactions with peers as well as changes in social contexts, financial situation, and living conditions [8,62]. In particular, students seem to be challenged by managing a new independent life and financial difficulties [63,64], poor housing conditions [65,66], and lack of social support [67,68,69,70].

With respect to the wellbeing of young workers, it seems that working life can compromise physical health and healthy lifestyles, in particular in those who are not concurrently studying, suggesting a need for specific prevention programs addressed to this population. Indeed, it seems that attending undergraduate or postgraduate courses constitutes a protective factor for healthy habits among emerging adults, whether or not they are also engaged in a working activity. Moreover, research has suggested that the interpretation of workers’ mental health is multifaceted. Sometimes, young workers struggle in recognizing the symptoms of poor mental health [14,71]. However, it is also possible that young workers experience lower levels of distress because they live a more rewarding EA than that of students. Crocetti and colleagues compared the experiences of Italian young adults and detected several differences in the perception of EA between students and workers [72]. The latter showed a lower need to explore possible alternatives among identities than that of students, and they felt less “in between”, perhaps because entering the workforce is a fundamental achievement towards an adult life. Simultaneously, they felt they had fewer possibilities open to them because, by choosing a job, they had turned down other possible choices. Conversely, students showed a greater perception of instability and lower self-focus, perhaps because they: (i) questioned the value of their academic curricula as a means of independence and social mobility; (ii) experienced greater uncertainty about their future lives.

The analysis stratified by the year of the NHS (2005 or 2013) did not show relevant differences between these two periods. Nevertheless, the risk of weak mental health was slightly higher among students and working students than that in workers in 2013 compared with that observed in 2005. These results merit further investigation because there is evidence from previous global financial crises (as well as the one in 2008) linking these consequences to depression, anxiety, and poor quality of life [73,74,75,76,77,78].

Finally, future research should address major changes in the living experience of students, workers and working students connected to the current COVID-19 pandemic. Indeed, the outbreak and the measures to contain it are having a great impact on general population worldwide. Uncertainty, loss of control, isolation, and worries about one’s own health and that of beloved ones often lead to mental health issues and psychological distress [79,80,81]. Previous research showed that emerging adults are very prone to experience psychological distress during the current pandemic, and they could be considered as a vulnerable group [82,83,84,85,86]. Indeed, even if young adults are at low risk of physical health complications from COVID-19, they may be distressed by the pandemic’s secondary consequences, including isolation, financial consequences and worries about the future.

### Limitations

Our study had three main limitations. First, health outcomes were assessed through self-reported medical diagnoses and by SF-12, and not through clinical interviews. Hence, there was a risk of non-differential misclassification of the outcomes investigated and consequent attenuation of the associations estimated. Second, the cross-sectional design of our study did not allow for causal inference, nor to follow changes in health over time. This limitation is in addition to general limitations on using cross-sectional studies, which involved different samples across time, although remaining representative of the whole population. Further longitudinal studies are needed. Third, potential confounders, such as social support, financial and living conditions, and the social background of the families of the young adults enrolled, were not examined in our study. Future research should take these aspects into account.

## 5. Conclusions

The present study is a first attempt to shed light on the differences between the psychological wellbeing of young students and young workers in Italy. Our results seem to demonstrate that emerging adults are a pivotal target group for prevention and early intervention, particularly because ~75% of mental-health conditions arise in the mid-twenties [87]. The difficulties emerging adults face are a matter of public concern arising from structural transformations in society [88]. Indeed, as noted by Tanner and Arnett [89], the transition from an industrial economy to an information-based economy has led to a deferral of transition to employment, marriage, and parenthood, which has modified the experience of EA. Thus, our results are of considerable importance for psychologists as well as educational and occupation-based institutions. Our data may have clinical implications for planning prevention programs and clinical interventions targeted specifically not only at this development stage, but on the specific declinations of EA according to academic and occupational conditions.

## Figures and Tables

**Table 1:** Descriptive statistics of sociodemographic variables stratified by workers, students, and working students.

Sociodemographic and Lifestyle Variables	Workers (*n* = 12,645)	Students (*n* = 5443)	Working Students (*n* = 524)	Total (*n* = 18,612)	*p*
Sex					≤0.001
Male (%)	7469 (59.1)	2402 (44.1)	222 (42.4)	10,093 (54.2)	
Female (%)	5176 (40.9)	3041 (55.9)	302 (57.6)	8519 (45.8)	
Age (years)					≤0.001
Median (IQR)	26 (23–28)	22 (21–25)	26 (23.5–28)	25 (22–27)	
Economic conditions					≤0.001
Good (%)	339 (2.7)	173 (3.2)	16 (3.0)	528 (2.8)	
Adequate (%)	8250 (65.3)	3682 (67.7)	375 (71.6)	12,307 (66.1)	
Scarce (%)	3508 (27.7)	1384 (25.4)	112 (21.4)	5004 (26.9)	
Totally inadequate (%)	548 (4.3)	204 (3.7)	21 (4.0)	773 (4.2)	
Year of survey					≤0.001
2005 (%)	8106 (64.1)	2272 (41.7)	295 (56.3)	10,673 (57.3)	
2013 (%)	4539 (35.9)	3171 (58.3)	229 (43.7)	7939 (42.7)	
BMI (kg/m^2^)					≤0.001
Underweight (<18.4)	836 (6.6)	515 (9.4)	56 (10.7)	1407 (7.5)	≤0.001
Normal (18.5–24.9)	9048 (71.6)	4261 (78.3)	382 (72.9)	13,691 (73.6)	
Overweight (25–29.9)	2357 (18.6)	593 (10.9)	76 (14.5)	3026 (16.3)	
Obese (>30)	404 (3.2)	74 (1.4)	10 (1.9)	488 (2.6)	
Current smoker					≤0.001
No	8381 (66.3)	4439 (81.6)	413 (78.8)	13,233 (71.1)	
Yes	4264 (33.7)	1004 (18.4)	111 (21.2)	5379 (28.9)	
Currently undertaking sporting activity					≤0.001
No	4865 (38.5)	1329 (24.4)	137 (26.1)	6331 (34.0)	
Yes	7780 (61.5)	4114 (75.6)	387 (76.9)	12,281 (66.0)	
Geographic region of Italy					≤0.001
Northern	6151 (48.7)	1735 (31.9)	250 (47.7)	8136 (43.7)	
Central	2230 (17.6)	963 (17.7)	114 (21.8)	3307 (17.8)	
Southern and Insular	4264 (33.7)	2745 (50.4)	160 (30.5)	7169 (38.5)	

IQR: Interquartile range.

**Table 2:** Descriptive statistics of health-outcome variables stratified by workers, students, and working students.

Health-Outcome Variables	Workers (*n* = 12,645)	Students (*n* = 5443)	Working Students (*n* = 524)	Total (*n* = 18,612)	*p*
Anxiety or depression					0.649
No (%)	12,518 (99.0)	5393 (99.1)	521 (99.4)	18,432 (99.0)	
Yes (%)	127 (1.0)	50 (0.9)	3 (0.6)	180 (1.0)	
Perceived health					≤0.001
Good	11,245 (88.9)	5019 (92.2)	462 (88.2)	16,726 (90.0)	
Poor	1400 (11.1)	424 (7.8)	62 (11.8)	1886 (10.1)	
MCS					≤0.001
Median (IQR)	53.5 (49–57.9)	53 (47.3–57.8)	52.5 (45.5–56)	53 (48.1–57.8)	
PCS					≤0.001
Median (IQR)	56.0 (54.7–57)	56.7 (55.7–57.1)	56.1 (54.9–57.1)	56.0 (55–57)	

MCS: Mental Component Score; PCS: Physical Component Score.

**Table 3:** Odds ratios for workers, students and working students after full adjustment of multivariate logistic regression models (sex, age, BMI, current sporting activity, current smoking, economic conditions, year of survey, and geographic region).

Predictors	Workers	Students	Working Students
Anxiety and depression	1	**1.48 (1.02–2.17)**	0.56 (0.18–1.76)
Poor perceived health	1	**0.87 (0.77–0.99)**	1.09 (0.83–1.43)
MCS < 40 (I decile)	1	**1.63 (1.45–1.83)**	**1.68 (1.31–2.17)**
PCS < 50.5 (I decile)	1	**0.72 (0.63–0.82)**	0.93 (0.69–1.23)

95% confidence intervals are shown in brackets. Significant results are in bold.

## Data Availability

The datasets generated for this study are available on request from the corresponding author.
